# Supplementary material for: Splice-Junction-Based Mapping of Alternative Isoforms in the Human Proteome
Source: Cell Rep. Author manuscript; Available in PMC 2020 Jan 15. (PMC6961840; doi:10.1016/j.celrep.2019.11.026)
Supplement: 2 [file NIHMS1546469-supplement-2.zip › DF1/PXD006675/LeftCoronaryArtery/LeftCoronaryArtery_2_TTN_APHVEFLRPLTDLQVR.pdf]

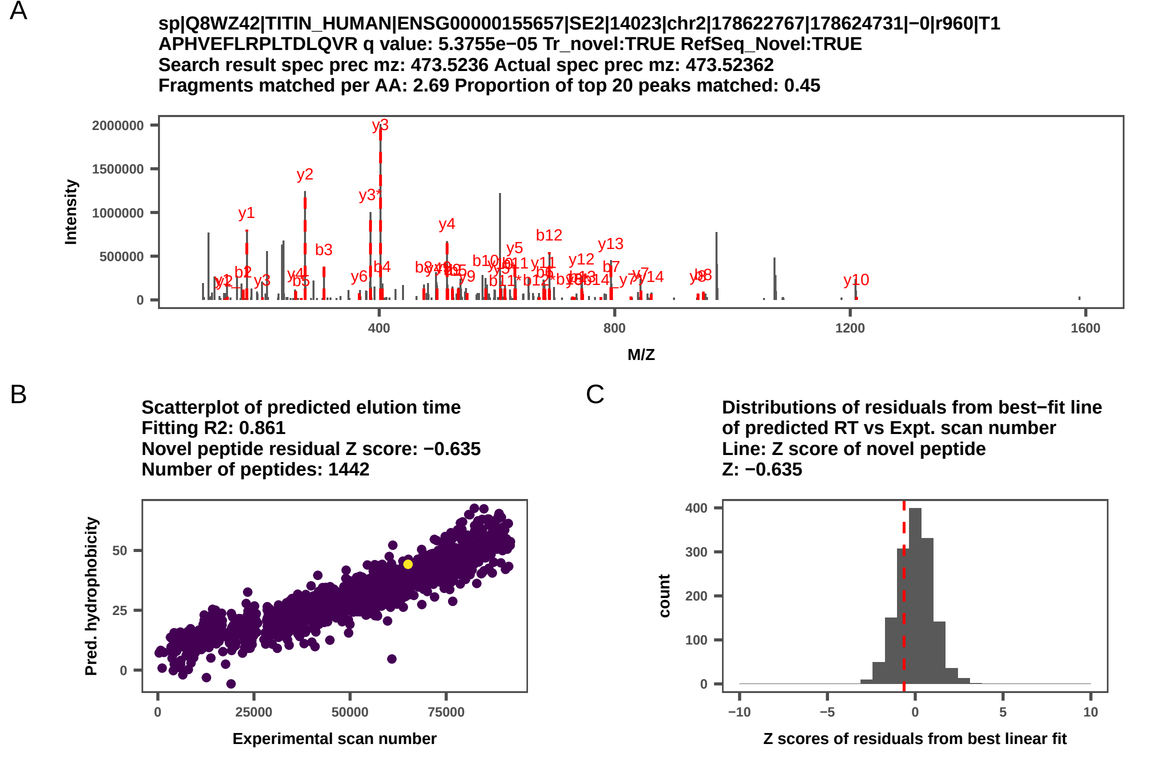
A
sp|Q8WZ42|TITIN_HUMAN|ENSG00000155657|SE2|14023|chr2|178622767|178624731|−0|r960|T1
APHVEFLRPLTDLQVR q value: 5.3755e−05 Tr_novel:TRUE RefSeq_Novel:TRUE
Search result spec prec mz: 473.5236 Actual spec prec mz: 473.52362
Fragments matched per AA: 2.69 Proportion of top 20 peaks matched: 0.45
Intensity
2000000
1500000
1000000
500000
0
M/Z
400
800
1200
1600
B
Scatterplot of predicted elution time
Fitting R2: 0.861
Novel peptide residual Z score: −0.635
Number of peptides: 1442
Pred. hydrophobicity
50
25
0
Experimental scan number
0
25000
50000
75000
C
Distributions of residuals from best−fit line
of predicted RT vs Expt. scan number
Line: Z score of novel peptide
Z: −0.635
count
400
300
200
100
0
Z scores of residuals from best linear fit
−10
−5
0
5
10
